# Incidence of Hippocampal Metastases: Laterality and Implications for Unilateral Hippocampal Avoiding Whole Brain Radiotherapy

**DOI:** 10.1155/2018/2459608

**Published:** 2018-12-13

**Authors:** Tomas Kazda, Adela Misove, Petr Burkon, Petr Pospisil, Ludmila Hynkova, Iveta Selingerova, Adam Dziacky, Renata Belanova, Martin Bulik, Zdenek Rehak, Alexandr Poprach, Ondrej Slama, Pavel Slampa, Ondrej Slaby, Radim Jancalek, Radek Lakomy

**Affiliations:** ^1^Department of Radiation Oncology, Masaryk Memorial Cancer Institute, Zluty kopec 7, 656 53 Brno, Czech Republic; ^2^Department of Radiation Oncology, Faculty of Medicine, Masaryk University, Kamenice 5, 625 00 Brno, Czech Republic; ^3^Central European Institute of Technology, Masaryk University, Kamenice 5, 625 00 Brno, Czech Republic; ^4^Department of Paediatric Haematology and Oncology, 2nd Faculty of Medicine, Charles University in Prague and Motol University Hospital, V Uvalu 84,150 06 Praha 5, Czech Republic; ^5^Faculty of Medicine, Masaryk University, Kamenice 5, 625 00 Brno, Czech Republic; ^6^Regional Centre for Applied Molecular Oncology (RECAMO), Masaryk Memorial Cancer Institute, Zluty kopec 7, 656 53 Brno, Czech Republic; ^7^Department of Radiology, Masaryk Memorial Cancer Institute, Zluty kopec 7, 656 53 Brno, Czech Republic; ^8^Department of Diagnostic Imaging, St. Anne's University Hospital Brno, Pekarska 53, 656 91 Brno, Czech Republic; ^9^Department of Diagnostic Imaging, Faculty of Medicine, Masaryk University, Kamenice 5, 625 00 Brno, Czech Republic; ^10^Department of Comprehensive Cancer Care, Faculty of Medicine, Masaryk University, Kamenice 5, 625 00 Brno, Czech Republic; ^11^Department of Comprehensive Cancer Care, Masaryk Memorial Cancer Institute, Zluty kopec 7, 656 53 Brno, Czech Republic; ^12^Department of Nuclear Medicine and PET Center, Masaryk Memorial Cancer Institute, Zluty kopec 7, 656 53 Brno, Czech Republic; ^13^Department of Neurosurgery-St. Anne's University Hospital Brno, Faculty of Medicine, Masaryk University, Kamenice 5, 625 00 Brno, Czech Republic; ^14^Department of Neurosurgery, St. Anne's University Hospital Brno, Pekarska 53, 656 91 Brno, Czech Republic

## Abstract

**Introduction:**

Hippocampi sparing whole brain radiotherapy (WBRT) is an evolving approach in the treatment of patients with multiple brain metastases, pursuing mitigation of verbal memory decline as a consequence of hippocampal radiation injury. Accumulating data are showing different postradiotherapy changes in the left and right hippocampus with a theoretical proposal of only unilateral (dominant, left) hippocampal sparing during WBRT.

**Method:**

The aim of this retrospective study is to describe spatial distribution of brain metastases on MRI in a cohort of 260 patients (2595 metastases) and to evaluate distribution separately in the left and right hippocampus and in respective hippocampal avoiding zones (HAZ, region with subtherapeutic radiation dose), including evaluation of location of metastatic mass centre.

**Results:**

The median number of brain metastases was three, with lung cancer being the most common type of primary tumour; 36% had single metastasis. Almost 8% of patients had metastasis within hippocampus (1.1% of all metastases) and 18.1% of patients within HAZ (3.3% of all metastases). No statistically significant difference was observed in the laterality of hippocampal involvement, also when the location of centre of metastases was analyzed. There were more patients presenting the centre of metastasis within left (15) versus right (6) HAZ approaching the borderline of statistical significance.

**Conclusion:**

No significant difference in the laterality of BM seeding within hippocampal structures was observed. The hypothesized unilateral sparing WBRT would have theoretical advantage in about 50% reduction in the risk of subsequent recurrence within spared regions.

## 1. Introduction

The paradigm of palliative radiotherapy of brain metastases (BM) has been recently shifting towards strengthen the quality of life (QoL), especially neurocognitive functions [1, 2]. For better preservation of neurocognition, stereotactic radiotherapy has become the currently recommended approach both in upfront treatment of limited brain metastases [3, 4] as well as in postoperative adjuvant radiotherapy [5, 6]. Apart from local stereotactic radiotherapy, many other strategies, including administration of N-methyl-D-aspartate receptor antagonist memantine, are being investigated in order to mitigate the well-known adverse iatrogenic effects of classical whole brain radiotherapy (WBRT), which has been utilized for decades as a simple, cheap, and widely available treatment of BM [7–10]. Hippocampal sparing during WBRT is a recent modification of radiotherapy that provides a low risk of adverse events with appropriate local and distal brain control [11].

Radiation injury (radioinjury) of the hippocampus is a phenomenon described from preclinical experiments and clinical observations, with radiotherapy doses as low as 2 Gy leading to changes in neural progenitor cells residing within hippocampal neurogenic niches and being involved in memory formation [11–14]. Following promising results from the single-arm phase II clinical trial RTOG 0933, in which conformal avoidance of both hippocampi during WBRT was associated with preservation of memory and QoL compared to historical controls [14], the randomized phase III trial of WBRT combined with memantine and with or without hippocampal sparing is currently evaluating the potential of hippocampal sparing for patients with brain metastases (NRG CC001-NCT02360215).

What is missing is a clear understanding of eventual laterality of hippocampal radioinjury. Some preliminary evidence, however, suggests differential changes in the left and in the right hippocampus after radiotherapy or surgery [15–20]. In our previous prospective study, we observed a correlation between post-WBRT verbal memory impairment and changes in the left hippocampus measured by* in vivo* magnetic resonance spectroscopy, whereas no such correlation was observed in the right hippocampus [19]. In another recent study focused on hippocampal radiation dose volume effects and memory deficits, in which combined data from another three prospective studies were analyzes, the left hippocampus appeared more sensitive to radiation than the right one [20]. For example, a 20% risk of decline in verbal memory was associated with the maximal delivered dose of 28.8 Gy to the right hippocampus, but with only 23.7 Gy to the left one. Considering that post WBRT cognitive impairment (represented mainly by verbal memory deficits) would have been associated predominantly with unilateral hippocampal radioinjury, only unilateral (left) sparing during WBRT may be judged. This novel approach of unilateral hippocampus sparing WBRT would be associated with reduced concern regarding subtherapeutic dose in spared regions and with the possibility of improved sparing of single hippocampus. In our pilot dosimetric study, left unilateral sparing yielded lower doses in spared hippocampus with a more homogenous irradiation of the remaining brain [21]. To justify clinical testing of this radiotherapy technique, it would be useful to evaluate whether there is some difference in the incidence of BM within left versus right hippocampus; however, no prior study has assessed the laterality of hippocampal BM seeding.

The aim of this retrospective study is to describe spatial distribution of BM in a cohort of 260 consecutive patients with a total of 2595 BM, evaluating distribution separately in the left and the right hippocampus and in respective hippocampal avoiding zones (HAZ).

## 2. Material and Methods

### 2.1. Patients and Image Selection

Consecutive patients with newly diagnosed BM referred between 1.1.2011 and 31.12.2014 to radiotherapy at Masaryk Memorial Cancer Institute in Brno, Czech Republic, were included in this retrospective study. Patients with available MRI scan that revealed first BM were eligible for further analysis. This MRI was used to describe spatial distribution of BM in order to avoid bias of previous local treatment of BM in estimation of incidence of hippocampal BM. Basic clinical data was obtained from electronic medical records. All patients signed the informed consent allowing usage of their clinical and imaging data for research purposes in an anonymous form.

### 2.2. Image Analysis

**Figure 1:**
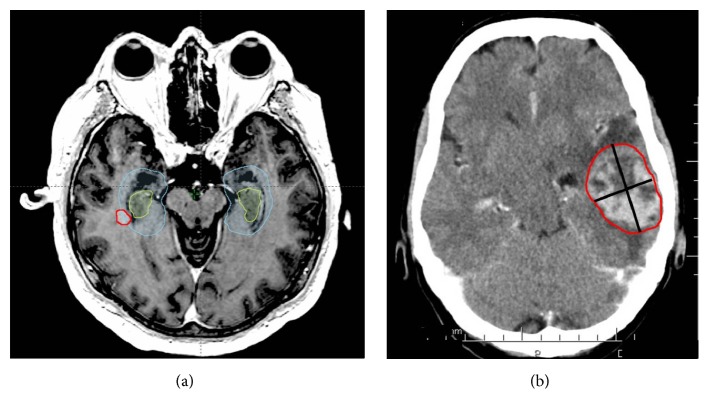
Illustrative cases of contouring and evaluation of hippocampal metastasis (a). Large metastasis (b) touching the hippocampus at the border illustrates the need for an analysis of the centre of the mass to enable a valid assessment of spatial relationships to potentially undertreated perihippocampal zones.

The location of BM was described in the first instance as temporal, occipital, parietal, frontal, or other (cerebellum, brain stem, diencephalon, or leptomeningeal BM) and BM were subsequently quantified within each region. MRI scans (T1 weighted sequence with intravenous administration of contrast agent) were transferred and imported into Eclipse™ radiotherapy treatment planning system (Varian Medical Systems, Palo Alto, CA), which enables smart contouring tools such as individual structures segmentation, isotropic expansions, and several Boolean operations. Left (LH) and right hippocampi (RH) were separately contoured in all patients referring to RTOG contouring atlas (Hippocampal contouring: a contouring Atlas for RTOG 0933) [22]. An expansion of 5 mm was performed to create left and right HAZ [14, 23]. All BM in the proximity of HAZ were manually contoured. The centre of all metastases (as the initial focal point of metastatic settlement) was also spatially correlated to both hippocampi and HAZ. All structures were double-checked and approved by experienced radiologists. Intersections of contoured BM and LH, RH, and pertinent HAZ were analyzed using Boolean operators (Figure 1).

### 2.3. Statistical Analysis

Basic statistics were employed to describe initial patients' characteristics. Binominal test was used for the calculation of the difference between BM occurrence within LH, RH, and pertinent HAZ. The evaluation of different number of metastases in the right and left hippocampus (or HAZ) required a comparison of patients who had more metastases in the right and in the left side. All significance testing was performed at the 0.05 level; R software version 3.2.4. was used for all analyses.

## 3. Results

### 3.1. Patient Characteristics

A total of 495 patients were screened for eligibility; 260 (55%) had available MRI and were eligible for further BM analysis (total number of 2595 BM). The median number of BM was 3 with lung cancer being the most common type of primary tumour (120/260 patients; 46.2%). Thirty-six percent had single lesion. The other basic clinical characteristics are summarized in Table 1.

### 3.2. Spatial Distribution of Brain Metastases and Relation to Hippocampi

Spatial distribution of BM is summarized in Table 2. Most patients presented with frontal lobe BM (154/260 patients; 59.2%). The most common localization of BM was also frontal lobe with mean 6.25 BM and median 2 BM within frontal lobes. Within left and right temporal lobes, there was a mean of 1.48 and 2.57 BM and median of 1 and 1 BM. Eight percent of patients (20/260 patients) had BM within hippocampi and 18% of patients (47/260 patients) within HAZ. There was no statistically significant difference in the number of patients who had more BM in the right (9 patients) versus the left (8 patients) hippocampus. Similarly, no significant difference was observed in the number of patients with involvement of right (20 patients) versus left (22 patients) HAZ (p = 0.88). There was also no difference in the number of BM within right and left hippocampus (p = 0.57) or within right and left HAZ (p = 0.91).

Furthermore, the presence of centre of a mass of BM within hippocampi and HAZ was evaluated. Five patients (5/260; 1.9%) developed BM whose centre was located within hippocampus and 9.6% of patients (25/260 patients) within HAZ. Higher number of patients developed more metastases whose centre was within left HAZ (15 patients) comparing to right HAZ (6 patients), approaching the borderline of statistical significance with p = 0.07. Four patients had the same number of metastases within left and right HAZ. Further details are summarized in Table 3.

## 4. Discussion

No significant difference in the laterality of BM seeding within hippocampus was observed in this large retrospective study. Nevertheless, normative standards are not known and, thus, before the start of this study, it was not possible to estimate the sample size needed to reach statistical significance with sufficient power. However, we believe that the lack of significance that we observed in the analysis of 260 patients indicates that there is truly no difference in the laterality of hippocampal BM involvement.

Several previous retrospective studies described perihippocampal incidence of BM [24–30]. Earlier studies focused on a general estimation of perihippocampal BM incidence, while further trials aimed at assessing some specific aspect such as the measurement of the distance of BM from hippocampi in Harth* et al.* [28] or the determination of the number of brain metastases in patients with melanoma [29] or breast cancer [30]. Nevertheless, no studies have specified laterality of BM location. Data from all previous studies indicate that hippocampi are a rare site of BM and that hippocampus avoiding WBRT is a safe procedure with low risk of undertreatment in hippocampi or HAZ. A construction of HAZ during radiotherapy planning is needed to generate a dose gradient fallout from the surrounding brain (irradiated to the full prescribed dose), but delivered dose within HAZ might be insufficient to control micrometastases.

The ideal methodology for assessing this risk of hippocampal (or HAZ) metastases would be a close observation of patients with BM treated with HA-WBRT and followed with regular imaging. Considering the relatively low incidence of hippocampal metastases in general, a high number of patients treated by this complex RT technique would be needed to report data with sufficient power. Even more patients would be needed considering the aim of our study to describe the potential difference in laterality of hippocampal metastatic seeding (what means to divide enrolled patients into cohorts). Given the current absence of such a dataset in available literature before ongoing trials will be published, the retrospective review of large cohort of radiotherapy-naïve patients was chosen as the best possible approach currently to estimate the hippocampal and HAZ metastases incidence before eventual initiation of trials of unilateral hippocampal sparing. This approach of using treated patient data retrospectively for an analysis also seems to be ethically preferable comparing to the straightforward treatment of patients with unilateral hippocampal sparing WBRT and the evaluation of the development of BM in spared region in follow-up. With this retrospective description of the distribution of metastases, it can be at least estimated what is the risk of hippocampal (or HAZ) failure after HA-WBRT. Recently, in a pooled analysis of available data, we summarized the incidence of BM in 1557 patients from listed studies [24–30] and calculated that BM is present within hippocampi in 1.6% of patients and within HAZ in 9%, representing 0.6% and 2.8% of all BM, respectively [31]. In comparison to this pooled analysis, we observed in the current study a much higher incidence of BM within hippocampus (in 7.7% patients representing 1.1% of all BM) as well as in HAZ (in 18.1% of patients representing 3.3% of all BM). This higher incidence observed in our unselected real-practice cohort may be explained by a relatively high mean number of BM (mean number of 10 BM compared to a mean of 4.5 BM in pooled analysis) with many patients presented initially with multiple BM disease. In another recent descriptive analysis of consecutive series of 2419 patients with BM treated at the Medical University of Vienna between 1990 and 2011, 48.7% of patients presented with a singular BM, 27.7% with 2–3, and 23.5% with >3 BM [32]. The corresponding percentages in our current study are as follows: 36%, 22%, and 42%, respectively. Some patients are referred to our radiotherapy department from relatively distant tertiary outpatient oncology practices, some with less availability and throughput of MRI devices, potentially explaining the lower number of limited brain disease in our cohort that contributed to relatively higher hippocampal involvement. Regardless of a possibly high incidence of BM observed in our study, progression in the HAZ area was very rare in patients from the prospective RTOG 0933 trial, which reported progression within HAZ in only 3 out of 67 progressed patients (4.5%) after a radiotherapy performed with sparing of both hippocampi [14]. Altogether, despite these promising safety profile of RTOG 0933 and despite reported cases of advantageous usage of hippocampal sparing in real clinical practice, some controversy remains, especially regarding the safety of this method with concerns about undertreatment of HA regions [33, 34]. The superiority of HA-WBRT in the management of patients with BM and the potential update in current standards of care will need to be confirmed within prospective randomized trials, most notably in the above-mentioned NRG CC001-NCT02360215 trial or NRG-CC003-NCT02635009 (A Randomized Phase II/III Trial of Prophylactic Cranial Irradiation with or without Hippocampal Avoidance for Small Cell Lung Cancer). In the meantime, this approach should be considered experimental and utilized only in individually selected cases with a required planning MRI for hippocampi contouring as well as for the exclusion of potential small BM within hippocampi or HAZ [34].

The analysis presented here is the first large study focused on the laterality of hippocampal BM involvement. It contributes to the field by providing important knowledge needed before initiating programs of unilateral (left) hippocampus sparing during WBRT, which may be sufficient to preserve verbal memory, the most commonly affected neurocognitive domain following brain irradiation. Of course, evaluation of general QoL and of another neurocognitive domains related to the right (nondominant) side of brain would be needed in the trial where bilateral versus unilateral hippocampal sparing during WBRT would be tested. With the hypothesized noninferiority of unilateral sparing, there would be a possible advantage of about 50% reduction in the risk of subsequent recurrence within spared regions based on our current observation of no significant difference in the laterality of BM seeding. Moreover, unilateral hippocampal avoiding leads to dosimetrically increased sparing of preserved hippocampus [21].

We acknowledge several limitations of our study apart from its self-limiting retrospective nature, disallowing for example standardization of MRI, which should be ideally volumetric and standardized at baseline in order not to miss small lesion. The other limitations are mitigated by the analysis of location of centre of BM. With the generally spherical shape of BM, the centre of BM represents the site of initial focal growth of a micrometastasis. Whether this centre is placed within hippocampi or HAZ, in this particular patient, the focal point of metastatic settlement is inside the part of brain which would be spared in HA treatment approach (for example in previous HA prophylactic brain irradiation). On the other hand, the patient with a large metastasis within the left temporal lobe presented in the Figure 1 would be also classified as hippocampal since the border of BM touches the edge of the hippocampus. However, the spot where its centre is located is clearly outside the eventually undertreated HAZ and would receive a full dose of radiation (for example in previous HA prophylactic brain irradiation). Metastases in both outlined cases can be labelled as “hippocampal” but with different consequences behind, resolved by the analysis of the location of centres of metastases. Thus, discrimination between the centre of a mass and the border of a mass is necessary to comprehensively assess the risk of undertreating patients with HA-WBRT and would provide additional information in other ongoing trials as well. In our analysis of centres of BM, we observed interesting difference in patients with different number of centres of MTS in the right and left sides, where more patients presented with the centre of BM within left versus right HAZ (15 versus 6 patients; p = 0.07). These patients warrant further study including the analysis of potential changes in cerebral blood supply. Thus, the most reliable feature of our study is probably this analysis of the centre of BM mass.

In conclusion, a relatively high incidence of perihippocampal BM was observed in our large retrospective study with no clear difference in the laterality of BM seeding within the hippocampus. Spatial patterns of failure in ongoing phase III trials, where the role of hippocampal avoiding WBRT is being assessed, may reveal true laterality of hippocampal recurrence rate and further support the proposed testing of unilateral hippocampal sparing during WBRT.

## Figures and Tables

**Table 1:** Basic clinical characteristics of included patients.

**Characteristic**	**N = 260**
**Age **(mean; years)	57.8
**Sex **(men; %)	125 (48.1%)
**Primary diagnosis**	
NSCLC	79 (30.4%)
SCLC	34 (13.1%)
Lung-not verified	7 (2.7%)
Breast	48 (18.5%)
Melanoma	30 (11.5%)
GYN	13 (5.0%)
Unknown origin	11 (4.2%)
RCC	11 (4.2%)
GI	9 (3.5%)
GU	5 (1.9%)
others	13 (5.0%)
**Initially disseminated**	96 (36.9%)
**Number of MTS**	
Mean	10.0
Median	3
IQR (25-75 %)	1-7

MTS: metastasis, NSCLC: non-small-cell lung cancer, SCLC: small-cell lung cancer, GI: gastrointestinal, GYN: gynecology, RCC: renal cell carcinoma, GU: genitourinal, and IQR: interquartile range

**Table 2:** Spatial distribution of brain metastases.

**Location of metastasis**	**N= 260 patients**
	**N= 2595 metastases**
**Temporal** – No. of patients	95 (36.5%)
Left	61 (23.5%)
Right	62 (23.9%)
**Temporal** – No. of metastases	310 (11.9%)
Mean/ Median/ IQR (25-75 %)	3.26/1/1-2.5
Left/Right	
Mean	2.48/2.57
Median	1/1
IQR (25-75 %)	1-2/1-2
**Occipital** – No. of patients	101 (38.9%)
Left	73 (28.1%)
Right	57 (21.9%)
**Occipital** – No. of metastases	288 (11.1%)
Mean/ Median/ IQR (25-75%)	2.85/1/1-2
Left/Right	
Mean	1.95/2.56
Median	1/1
IQR (25-75 %)	1-2/1-3
**Parietal** – No. of patients	128 (49.2%)
Left	85 (32.7%)
Right	83 (31.9%)
**Parietal** – No. of metastases	397 (15.3%)
Mean/ Median/ IQR (25-75 %)	3.10/1/1-2
Left/Right	
Mean	2.55/2.17
Median	1/1
IQR (25-75 %)	1-2/1-2
**Frontal** – No. of patients	154 (59.2%)
Left	121 (46.5%)
Right	98 (37.7%)
**Frontal** – No. of metastases	962 (37.1%)
Mean/ Median/ IQR (25-75 %)	6.25/2/1-4
Left/Right	
Mean	4.02/4.86
Median	1/2
IQR (25-75 %)	1-2/1-4
**Other locations** – No. of patients	29 (11.2%)
**Other locations **– No. of metastases	638 (24.6%)

Abbreviations: No.- number, MTS- metastasis, IQR-interquartile range

**Table 3:** Presence of brain metastases (or their centre) within right and left hippocampus and hippocampal avoiding zones and laterality of hippocampal involvement.

	within	within	within	P value	within	within	within	P value
	H	left H	right H		HAZ	left HAZ	right HAZ	
**Patients (n=260) with edge of MTS**	20 (7.7%)	12 (4.6%)	12 (4.6%)		47 (18.1%)	30 (11.5%)	27 (10.4%)	
**Patients (n=260) with *more* MTS**		8	9	NS		22	20	p = 0.88
**Patients (n=260) with centre of MTS**	5 (1.9%)	3 (1.2%)	3 (1.2%)		25 (9.6%)	19 (7.3%)	12 (4.6%)	
**Patients (n=260) with *more* centre of MTS**		2	2	NS		15	6	p = 0.07
**Number of MTS (n=2595) with edge**	28 (1.1%)	12 (0.5%)	16 (0.6%)	p = 0.57	86 (3.3%)	42 (1.6%)	44 (1.7%)	p = 0.91
**Number of MTS (n=2595) with ** **centre of MTS**	7 (0.3%)	3 (0.1%)	4 (0.1%)	NS	41 (1.6%)	25 (0.9%)	16 (0.6%)	p = 0.21

MTS: metastasis, H: hippocampus, HAZ: hippocampus avoiding zone, NS: not specified, and p value close to 1.

## Data Availability

The data used to support the findings of this study are available from the corresponding author upon request.
